# A new screening method for periodontitis: an alternative to the community periodontal index

**DOI:** 10.1186/s12903-016-0216-x

**Published:** 2016-06-02

**Authors:** Yoshiaki Nomura, Ayako Okada, Erika Kakuta, Takahide Gunji, Seiji Kajiura, Nobuhiro Hanada

**Affiliations:** Department of Translational Research, Tsurumi University School of Dental Medicine, 2-1-3 Tsurumi, Tsurumi-ku, Yokohama, 230-8501 Japan; Shimane Dental Association, 141-9 Minamitamachi, Matsue, Shimane 690-0884 Japan; Department of Health and Welfare, Shimane Prefectural Government, 1 Tonomachi, Matsue, Shimane 690-8501 Japan

**Keywords:** Periodontitis, Screening, Community Periodontal Index, Saliva test

## Abstract

**Background:**

Periodontal screening plays an important role in the prevention of periodontal disease and promotes an improvement in oral health-related quality of life. The World Health Organization’s Community Periodontal Index should be carried out by well-trained dentists. However, the Community Periodontal Index is an invasive technique, and if used for periodontal screening, increases the cost of evaluation. In order to overcome these issues, we developed saliva tests for periodontal screening. The purpose of this study was to calculate the sensitivity and specificity of our method for measuring hemoglobin and lactate dehydrogenase levels in saliva.

**Methods:**

Inclusion criteria were adults aged over 20 years with at least 20 teeth remaining. The study population comprised 38 men and 54 women with a mean age of 50.03 years. Oral examinations were carried out by dentists, and the number of remaining teeth, presence or absence of calculus, bleeding on probing and pocket depth were recorded. In this study, periodontitis was defined according to the criteria of the Center for Disease Control and Prevention in partnership with the American Academy of Periodontology. In order to examine hemoglobin and lactate dehydrogenase levels in saliva, participants were instructed to chew on a standard-sized tasteless and odorless gum base for 5 min, during which time, stimulated whole saliva was continuously collected.

**Results:**

The sensitivity and specificity for hemoglobin levels were 0.759 and 0.763, respectively, and 0.722 and 0.711, respectively, for lactate dehydrogenase levels. Combining these two tests, when samples tested positive for both hemoglobin and lactate dehydrogenase, the positive predictive value was 91.7 %.

**Conclusion:**

Measuring hemoglobin and lactate dehydrogenase levels in saliva is a less invasive method than the Community Periodontal Index. Therefore, our saliva tests may be a viable alternative to the Community Periodontal Index for periodontal screening.

## Background

Periodontal disease is one of the most prevalent oral diseases among the middle-aged and elderly population. With the advance of clinical knowledge and techniques, early detection can prevent the progression of periodontal disease. Therefore, periodontal screening may help prevent periodontal disease and improve oral health-related quality of life. The Community Periodontal Index (CPI), which was originally developed by the World Health Organization to measure community oral health, is commonly used for periodontal screening. However, the CPI does have some fundamental biological problems. For example, the CPI requires probing, which puts patients with periodontitis at risk of bacteremia [1]. The prevalence of odontogenic bacteremia is more than 30 % [2]. Therefore, the CPI should only be used to measure community oral health status in small populations. In addition, the CPI only evaluates index teeth; missing index teeth makes precise comparisons with other data difficult. Furthermore, well-trained dentists are necessary as examiners, and this adds to the cost of evaluation. Although several periodontal indices have been developed, only the CPI has been applied in mass checkups in Japan.

The Industrial Safety and Health Act stipulates that Japanese companies must offer annual medical checkups for all employees. Almost all medical checkups are carried out under a mass checkup system in which dental checkups are optional. However, cost and time are important factors for Japanese companies, and dentists can only check a limited number of subjects, so few companies choose to cover dental checkups.

Saliva tests developed for the screening of periodontal disease can help overcome these problems. The cost of saliva tests is reasonable because conventional markers used for routine health checkups are applied. The cost for one sample is about 10 USD. In addition, saliva tests are less invasive than the CPI, and the collection of saliva samples can be performed by non-specialized dental staff. An association between salivary levels of hemoglobin (Hb) and gingival inflammation has been reported [3], and a test paper strip method developed to detect Hb in saliva has been shown to be a useful screening test for periodontal diseases [4]. In addition, lactate dehydrogenase (LD) activity in saliva may constitute a specific indicator of oral mucosal lesions with tissue breakdown, including periodontal disease [5, 6].

Salivary levels of Hb and LD have shown significant relationships with the CPI and probing pocket depth (PD) [7–11], and salivary levels of LD can be a predictive marker of healthcare costs [12].

Traditionally, a colorimetric detection method was used to measure Hb, but this method could only detect free Hb [7, 9, 11]. Therefore, Hb derived from food such as meat or fish was included, making the diagnostic precision of Hb levels inadequate. Methods for measuring Hb have since been improved. Currently, two reagents for the stool occult blood test are commercially available. These reagents, which use polyclonal antibodies and employ tracers such as colloidal gold labels or latex beads, can be applied to measure salivary Hb. This method enables more sensitive detection of Hb in saliva [8, 12, 13]. However, no studies have been conducted regarding the use of this improved method for periodontal screening.

Although this saliva test is related to the CPI and PD, whether it is more suitable than the CPI for screening periodontal disease remains unclear. Therefore, the objective of this study was to compare the sensitivity and specificity of the saliva test with those of the CPI for detecting periodontal disease, especially periodontitis, and to examine whether the cutoff values from these tests could be used as a screening tool for periodontal disease.

## Methods

### Study population

The study population was selected from patients who attended eight private dental clinics under the administration of the Shimane Dental Association in Japan. Patients older than 20 years who had more than 20 teeth remaining were included, and those who were missing any index teeth from the sextant classified by the CPI or who had associated lifestyle-related diseases were excluded. Smoking status, current medication and presence of lifestyle-related diseases were checked by patient interviews.

### Sampling frame and sample size calculation

An a priori power analysis was performed with an alpha value of 0.05 and a power of 0.80 to compare the patients with or without periodontitis and identify representative samples for the Japanese population. A two-way table was calculated to detect differences regarding positive or negative findings for LD in saliva. The data for this calculation were obtained from our previous report [9]. The minimum sample size in both the positive and negative groups to detect statistically significant differences in salivary levels of LD was 29. As previously described, the method used to measure Hb in this study was different from that used in our previous report. Therefore, the sample size was calculated for LD only. Based on a post-hoc power analysis, there were 18 and 26 patients in the Hb and LD groups, respectively.

Based on a survey of dental diseases conducted by the Japanese Ministry of Health, Labor and Welfare in 2005, 15.4 % of patients aged 40–44 years and 12.1 % aged 45–50 years have no symptoms involving the gums, which corresponds to CPI Code 0. Therefore, healthy patients within the range of 10–15 % were selected as controls. The goal was to enroll 10 patients as healthy controls and 30 as subjects with periodontal symptoms. All analyses were performed using S-plus software (version 6.1; NTT DATA, Tokyo, Japan).

### Clinical examination

Oral examinations were carried out by dentists (*n =* 8) at each of the dental clinics. Before the study began, CPI criteria were calibrated with a CPI probe. Each dentist examined three patients. Inter-examiner calibrations were satisfactory. Inter-examiner differences occurred in two cases; therefore, the kappa value range for intra-examiner calibration was from 1 to 0.22.

Each tooth was examined using the six-point method, and the number of remaining teeth, presence or absence of calculus, bleeding on probing (BOP) and PD were recorded. When periodontal pockets deeper than 4 mm were found in any of the index teeth, probing with the CPI probe was repeated for that tooth. Each patient was diagnosed according to CPI criteria from Code 0 to Code 4 (Code 0: health periodontal conditions; Code 1: gingival bleeding on probing; Code 2: calculus and bleeding; Code 3: periodontal pocket 4–5 mm; and Code 4: periodontal pocket ≥6 mm) [14, 15]. In this study, periodontitis was diagnosed according to the criteria of the Center for Disease Control and Prevention in partnership with the American Academy of Periodontology [16]. Briefly, patients with periodontal pockets in two or more interproximal sites with a clinical attachment level of ≥3 mm, in two or more interproximal sites with a PD ≥4 mm (for different teeth), or in one site with a PD ≥5 mm were diagnosed as having periodontitis. Clinical attachment levels were only measured when a periodontal pocket ≥3 mm was found at an interproximal site.

### Measuring salivary Hb and LD

Saliva samples were collected prior to the oral clinical examinations. According to the manufacturer’s instructions, saliva samples were collected at least 2 h after eating, drinking, or tooth brushing. Participants were instructed to chew on a standard-sized tasteless and odorless gum base for 5 min, during which time, stimulated whole saliva was continuously collected. To examine salivary levels of Hb, 100 μL of the collected saliva was immediately transferred into a diluent solution with preserving agents and kept at 4 °C. Surplus saliva for measuring LD was also kept at 4 °C. Salivary levels of LD and Hb were measured using commercially available kits (L type Wako LDH J; Wako Chemical Industry, Osaka, Japan, and OC-HEMODIA AUTO S; Eiken Kagaku, Tokyo, Japan) according to the manufacturer’s instructions [8, 12].

### Statistical analysis

The differences in Hb or LD levels and presence or absence of periodontitis compared with CPI criteria [16] were analyzed using the Kruskal-Wallis or Mann–Whitney *U* test. The cutoff values for saliva tests and the CPI were obtained using receiver operator characteristics (ROC) curves. Based on the literature [17, 18], diagnostic efficacy was calculated and represented as sensitivity, specificity, positive and negative predictive values and Youden’s index (Sensitivity + Specificity-1). The association between periodontitis and positive or negative results in the saliva tests were analyzed using Fisher’s exact test. The decision tree procedure creates a tree-based classification model that classifies cases into groups or predicts the values of a dependent variable based on the values of independent variables. To construct the diagnostic chart, classification and regression tree (CART) analysis, which constructs a decision tree, was applied. CART splits the data into segments that are as homogeneous as possible with respect to the dependent variable.

Statistical analyses were performed using IBM SPSS Statistics (Version 19.0; IBM SPSS, Tokyo, Japan).

## Results

The study population consisted of 38 men (41.3 %) and 54 women (58.7 %) with a mean age ± standard deviation (SD) of 50.03 ± 17.86 years (age range: 20–83 years). Sixteen (17.2 %) of the participants were current smokers.

A descriptive analysis of clinical markers and salivary levels of Hb and LD was then calculated against CPI criteria (Table 1). Dose–response relations were observed for all markets except for CPI 1 for BOP and Hb and CPI 0 for LD. All differences were statistically significant based on the Kruskal-Wallis test. Regarding the number of current smokers, there was 1 for CPI 0, 2 for CPI 1, 8 for CPI 3 and 5 for CPI 4. When current smokers were excluded, the same tendencies were observed and all differences remained statistically significant.

**Table 1:** Descriptive analysis of bleeding on probing (BOP), pocket depth (PD), and salivary levels of hemoglobin (Hb) and lactate dehydrogenase (LD) against Community Periodontal Index (CPI) criteria

CPI	0 (*n =* 12)	1 (*n =* 8)	2 (*n =* 12)	3 (*n =* 30)	4 (*n =* 30)	*P*
BOP	0.31 ± 0.60	7.86 ± 6.36	3.78 ± 10.19	15.62 ± 15.30	19.83 ± 18.78	<0.001
(%)	0.00 (0.00–0.67)	6.64 (1.92–15.03)	0.00 (0.00–2.87)	12.27 (2.86–24.17)	11.37 (3.67–36.71)	
PD	1.85 ± 0.23	1.88 ± 0.44	2.14 ± 0.35	2.73 ± 0.39	3.44 ± 0.63	<0.001
(mm)	1.97 (1.60–2.00)	1.71 (1.54–2.24)	2.10 (2.01–2.35)	2.68 (2.39–3.08)	3.36 (3.01–3.97)	
Hb	1.08 ± 2.51	26.39 ± 72.62	1.08 ± 1.68	6.67 ± 14.95	26.55 ± 48.57	<0.001
(μg/mL)	0.10 (0.10–1.15)	0.80 (0.25–1.65)	0.40 (0.10–1.20)	2.10 (0.93–3.40)	6.45 (0.73–29.03)	
LD	346.58 ± 306.73	163.75 ± 64.99	247.75 ± 109.66	376.90 ± 360.77	544.17 ± 358.74	<0.001
(IU/L)	279.00 (128.75–410.50)	157.50 (111.00–212.25)	233.50 (192.00–3111.75)	30800 (177.00–412.00)	451.50 (229.50–694.75)	

Data are expressed as mean ± standard deviation and median (25 %, 75 % percentiles). Dose response relations were observed except for CPI 1 of the BOP% and Hb and for CPI 0 of LD. All differences were statistically significant (Kruskal-Wallis test)

*BOP* bleeding on probing, *PD* pocket depth, *Hb* hemoglobin, *LD* lactate dehydrogenase, *CPI* Community Periodontal Index

One patient classified as CPI 0 had high LD levels (1,223 IU/L), and one patient classified as CPI 1 had high Hb levels (206.1 μg/mL). When these patients were excluded, the mean ± SD value for LD was 266.91 ± 140.36 for CPI 0, and the mean ± SD value for Hb was 1.90 ± 0.714 for CPI 1.

Next, a descriptive analysis of clinical markers and salivary levels of Hb and LD was calculated against the presence or absence of periodontitis (Table 2). Hb and LD levels were markedly higher in patients with periodontitis than in those without. These differences were statistically significant according to the Mann–Whitney *U* test.

**Table 2:** Descriptive analysis of BOP, PD, Hb and LD in participants with and without periodontitis

	Periodontitis		*P*
	− (*n =* 38)	+ (*n =* 54)	
BOP	5.95 ± 10.15	17.58 ± 17.58	<0.001
(%)	0.96 (0.00–6.45)	11.37 (3.25–27.77)	
PD	2.09 ± 0.43	3.13 ± 0.65	<0.001
(mm)	2.03 (1.73–2.33)	3.10 (2.60–3.48)	
Hb	6.23 ± 33.33	18.46 ± 38.61	<0.001
(μg/mL)	0.35 (0.10–1.15)	3.10 (1.43–18.83)	
LD	263.00 ± 196.64	482.96 ± 387.34	<0.001
(IU/L)	228.00 (153.75–321.75)	404.00 (222.50–523.50)	

Data are expressed as mean ± standard deviation and median (25 %, 75 % percentiles). Hb and LD salivary levels were significantly higher in participants with than without periodontitis (Mann–Whitney *U* test)

*BOP* bleeding on probing, *PD* pocket depth, *Hb* hemoglobin, *LD* lactate dehydrogenase

**Fig. 1:**
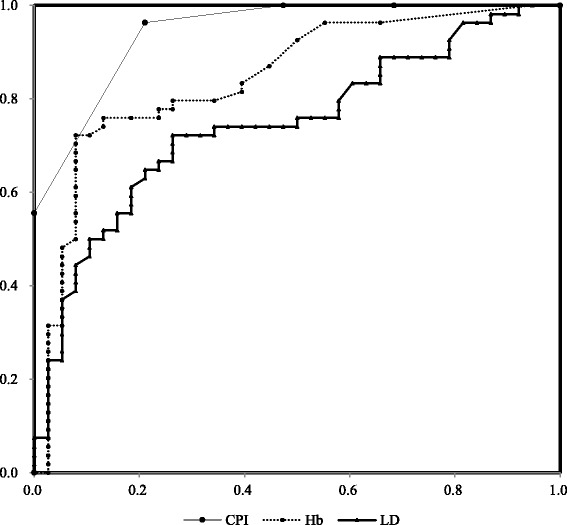
Receiver operator characteristics (ROC) curves for periodontitis based on the Community Periodontal Index (CPI) and salivary hemoglobin (Hb), and lactate dehydrogenase (LD) levels. The area under the ROC curve (AUC) was higher for the CPI than for the saliva tests (CPI, 0.954; Hb, 0.846; LD, 0.737)

Then, to compare the accuracy of the CPI and saliva tests in periodontal screening, we plotted ROC curves (Fig. 1) and calculated the sensitivities, specificities, positive and negative predictive values and Youden’s index for periodontitis (Table 3). Based on the ROC curves, we found that the area under the ROC curve (AUC) for the CPI was higher than that for the saliva tests (CPI: 0.954; Hb: 0.846; LD: 0.737). The optimal cutoff values for the CPI, Hb and LD were 3, 1.25 μg/mL and 298 IU/L, respectively.

**Table 3:** Community Periodontal Index (CPI) values and hemoglobin (Hb) and lactate dehydrogenase (LD) levels at screening

	Cutoff	*P*	Sensitivity	Specificity	Positive predictive value	Negative predictive value	Crude hit rate	Yorden’s index	AUC-ROC
CPI	3	<0.001	0.964	0.789	0.869	0.938	0.892	0.753	0.954
Hb (μg/mL)	1.25	<0.001	0.764	0.763	0.824	0.690	0.763	0.527	0.846
LD (IU/L)	298	<0.001	0.709	0.711	0.780	0.628	0.710	0.420	0.737

Sensitivities, specificities, positive and negative predictive values, Youden’s index and areas under the receiver operator characteristics curve (AUC-ROC) for periodontitis were calculated. *P* values were calculated using Fisher’s exact test

*Hb* hemoglobin, *LD* lactate dehydrogenase, *CPI* Community Periodontal Index

The CPI showed the highest values, and the AUC was 0.954. Based on the crude hit rate using with the CPI, 10 patients (10.1 %) were misdiagnosed as follows: two patients classified as CPI 2 had periodontitis, and eight patients classified as CPI 3 did not have periodontitis.

Using Hb and LD did not provide substantially more accuracy than the CPI; however, the sensitivity and specificity were more than 0.76 for Hb and more than 0.70 for LD, and AUC values were also high. Therefore, these markers appear to be applicable for periodontal screening.

**Fig. 2:**
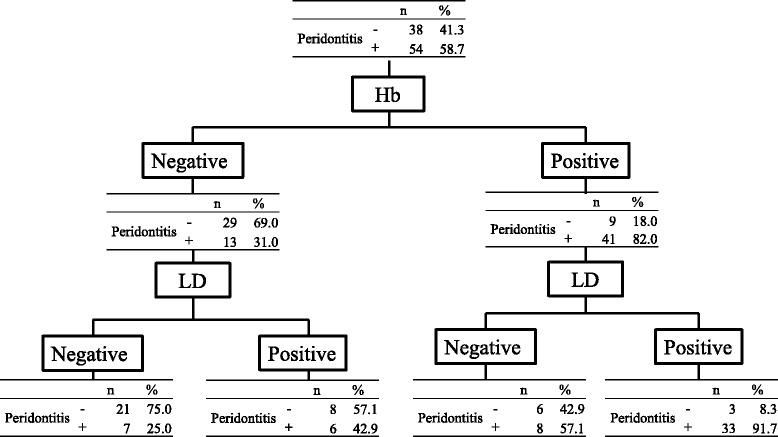
Diagnostic chart for periodontal screening by salivary Hb and LD levels. The cutoff points for Hb and LD were 1.25 μg/mL and 298 IU/L, respectively. When samples were positive for both Hb and LD, the positive predictive value was 91.7 %. When both Hb and LD were negative, the negative predictive value was 75.0 %

In addition, a diagnostic chart was constructed for Hb and LD (Fig. 2). Based on this chart, 91.7 % of patients testing positive for both Hb and LD had periodontitis, and 75 % of those testing negative did not.

## Discussion

In this study, pocket probing was carried out in dental clinics, and high sensitivity and specificity were obtained. Although the CPI is more accurate than salivary tests, it does have several drawbacks, including the fact that its accuracy may be adversely affected in mass screening. In addition, due to dental fees, the CPI is not cost-effective for mass screening, and the number of patients who can be examined by may be limited.

An accumulation of evidence regarding odontogenic bacteremia has also been reported. During dental procedures, and even during tooth brushing or mastication, oral bacteria and their components can easily disseminate into the systemic circulation. According to a systematic review by the American Academy of Orthopedic Surgeons and the American Dental Association, the prevalence of bacteremia by mastication is less than 5 %, and by pocket probing is more than 30 % [2]. In this respect, saliva tests are more advantageous than the CPI for mass screening.

Stimulated saliva samples were used in this study because resting saliva can be contaminated with blood from oral mucosal lesions. About 75 % of the LD in whole saliva originates from an extra-salivary gland source [19]. Therefore, saliva stimulated by chewing gum or paraffin measures biomarkers in the oral environment more accurately than resting saliva.

Some salivary biomarkers have been suggested to be associated with periodontal conditions. However, most of these biomarkers are limited to use in research. In addition, the cost for measuring these markers is high. As shown in Table 3, although the sensitivities and specificities for Hb and LD were moderate, they were higher than those for other biomarkers (gingival nitric oxide [10], sensitivity: 0.57, specificity: 0.94; blood IgG antibody titer against *Porphyromonas gingivalis* [20], Youden’s index: 1.36, AUC: 0.708). The sensitivities and specificities of Hb an LD were also higher than those obtained using self-administered questionnaires for the screening of periodontal disease [21–23] (sensitivity: 0.22, specificity: 0.81; sensitivity: 0.36, specificity: 0.97; sensitivity: 0.55, specificity: 0.98, respectively).

A dose–response relationship was observed in PD against the CPI. Patients classified as CPI 1 had higher BOP and Hb values than those classified as CPI 2. CPI coding is categorical, and thus sequential pathological conditions for periodontal disease are not coherent. CPI 1 indicates bleeding on probing, and this disease state easily increases salivary levels of Hb. CPI 2 is the presence of calculus, which is easily deposited on the lingual side of the lower anterior tooth. In some cases, calculus is deposited without gingival inflammation or mucosal damage. The results in this study regarding salivary Hb levels were similar to those reported using the test paper strip method (sensitivity: 0.752, specificity: 0.746) [4], suggesting that about 25 % of patients with periodontal disease have a small degree of gingival bleeding resulting from problems such as gingival overgrowth, and that healthy patients tend to have mucosal injuries other than periodontal disease.

The screening chart shown in Fig. 2 may facilitate screening-related decisions. In this chart, the positive and negative predictive values are important. If both Hb and LD are positive, the positive predictive value is 91.7 %. If both Hb and LD are negative, the negative predictive value is 75 %. High positive or negative predictive values can therefore be obtained using a combination of these saliva tests. Although the sensitivity and specificity of the Hb and LD saliva tests were inferior to those of the CPI, they appear to be adequate for the mass screening of periodontal disease.

## Conclusion

Measuring Hb and LD levels in saliva is a less invasive method than the CPI. The saliva tests may be a viable alternative to the Community Periodontal Index for periodontal screening.

## Abbreviations

AUC-ROC, Area under the receiver operator characteristics curve; BOP, Bleeding on probing; CART, Classification and regression trees; CPI, Community Periodontal Index; Hb, Hemoglobin; LD, Lactate dehydrogenase; PD, Pocket depth.
